# Prognosis of Pediatric Dilated Cardiomyopathy: Nomogram and Risk Score Models for Predicting Death/Heart Transplantation

**DOI:** 10.3390/children12070880

**Published:** 2025-07-03

**Authors:** Bowen Xu, Yue Yuan, Lu Gao, Zhiyuan Wang, Zhenyu Lv, Wen Yu, Hongfang Jin, Zhen Zhen, Zhihui Zhao, Jia Na, Aihua Hu, Yanyan Xiao

**Affiliations:** 1 Department of Cardiology, Beijing Children’s Hospital, National Center for Children’s Health, Capital Medical University, Beijing 100045, China; xubw2012@outlook.com (B.X.); yuanyuebj22@sina.com (Y.Y.); gaoluga33400122@163.com (L.G.); zhiyuanwang@ccmu.edu.cn (Z.W.); lzyedc@163.com (Z.L.); 15652639179@126.com (W.Y.); zhenzhen_bch@163.com (Z.Z.); huihui16166@sina.com (Z.Z.); najia198912@163.com (J.N.); 2 Department of Pediatrics, Peking University First Hospital, Beijing 100034, China; jinhongfang@bjmu.edu.cn; 3 State Key Laboratory of Vascular Homeostasis and Remodeling, Peking University, Beijing 100034, China; 4 Pediatric Chronic Disease Management Center, Beijing Children’s Hospital, National Center for Children’s Health, Capital Medical University, Beijing 100045, China; aihuacn@hotmail.com

**Keywords:** dilated cardiomyopathy, death, heart transplantation, predictive model, children

## Abstract

**Background:** This study aimed to develop a predictive model to assess risk factors and prognoses in pediatric patients with dilated cardiomyopathy (DCM). **Methods:** A total of 233 pediatric patients with DCM who were hospitalized between January 2019 and June 2024 were enrolled. The children were followed up and categorized into two groups: the death/heart transplantation (D/HT) group and the non-D/HT group. Univariate and multivariate analyses identified risk factors. A nomogram model and a scoring system were developed. The performance of these models was evaluated using the H-L test, ROC analysis, and internal validation. **Results:** The results demonstrated that the age of onset, cardiac functional classification III–IV, moderate-to-severe mitral regurgitation, low voltage in limb leads on an ECG, and the need for vasoactive drugs are independent predictors of D/HT risk in children with DCM. A nomogram model was developed, achieving an AUC of 0.804 (95% CI: 0.734–0.874), a sensitivity of 80.3%, and a specificity of 66.7%. A scoring system was established: 1 point for age of onset, 10 points for cardiac functional classification III–IV, 2.5 points for moderate-to-severe mitral regurgitation, 4 points for low voltage in limb leads on an ECG, 3 points for the need for vasoactive drugs, or 0 points if none of these criteria were met. When the cumulative score was ≥ 13.25, the sensitivity and specificity increased to 68.9% and 73.9%, respectively. **Conclusions:** We developed both a nomogram and a scoring system model, which are capable of rapidly and accurately predicting the risk of D/HT in children with DCM.

## 1. Introduction

Dilated cardiomyopathy (DCM) encompasses a heterogeneous group of myocardial disorders characterized by ventricular chamber dilation and impaired systolic function [1]. The prevalence of DCM in pediatric populations is estimated at 0.026% [2,3]. This condition is a leading cause of heart failure and sudden cardiac death, often necessitating heart transplantation as the definitive treatment [4]. Data from the National Heart, Lung, and Blood Institute indicate that approximately 46% of patients with DCM experience mortality or require heart transplantation within five years [5], a finding that is corroborated by multiple cohort studies [3,6]. Recent advancements have led to notable improvements in survival rates among pediatric patients with DCM [7]. However, high mortality and morbidity rates persist, underscoring the need for more effective risk stratification strategies [8,9]. Investigating prognostic factors in pediatric patients with DCM within the framework of current treatment guidelines holds substantial clinical significance. This study aims to identify and evaluate the risk factors influencing the prognosis of pediatric patients with DCM, thereby providing a robust reference for accurate risk stratification, prognostic assessment, individualized management, and tailored therapeutic strategies for this patient population.

## 2. Materials and Methods

### 2.1. Patients

This study conducted a retrospective analysis of 233 patients with DCM (119 males and 114 females, with a mean age of 61.41 ± 57.91 months) who were hospitalized in the Department of Cardiology, Beijing Children’s Hospital, Capital Medical University, China, between January 2019 and June 2024. This study was reviewed and approved by the Institutional Ethics Committee ([2025]-E-026-R).

Diagnostic criteria for DCM were as follows [10]: 1. left ventricular dilation, defined as a left ventricular end-diastolic diameter z-score of >2 when adjusted for body surface area (BSA), and 2. impaired systolic function, evidenced by echocardiographic parameters of an ejection fraction (EF) of <55% or a fractional shortening (FS) of <25%. To ensure the accuracy of the primary DCM diagnosis, relying solely on morphological imaging findings is insufficient, and the secondary causes of DCM must be rigorously excluded. Accordingly, patients with ischemic cardiomyopathy, arrhythmogenic cardiomyopathy, valvular heart disease, congenital heart disease, or cardiomyopathies secondary to systemic conditions (e.g., severe hepatic/renal dysfunction, hematologic disorders, connective tissue diseases, thyroid dysfunction, and malignancies) were excluded from this study, as were those receiving specific cardiotoxic medications.

### 2.2. Data Collection

To develop predictive models, we evaluated potential risk factors in accordance with the current guidelines [11]. Comprehensive medical histories were obtained from all pediatric patients upon admission. The data collected included the age at onset, sex, body mass index (BMI), presenting symptoms, cardiac functional classification, duration of heart failure, past medical history, and family history. For cardiac functional classification, children aged 5 years and older were assessed using the New York Heart Association (NYHA) functional classification system, while those younger than 5 years were evaluated using the Ross classification system [11]. Upon admission, venous blood samples were immediately collected for comprehensive hematological analysis. The panel of laboratory tests included a complete blood count (CBC), liver and renal function tests, electrolyte levels, myocardial enzyme profiles, serum thyroid function tests, serum homocysteine concentration, and serum 25-hydroxyvitamin D levels. Additionally, all pediatric patients underwent a 12-lead electrocardiogram (ECG) and 24 h Holter monitoring. The mean heart rate and heart rate variability over the 24 h period were meticulously recorded for further analysis.

All pediatric patients underwent echocardiography, and the following parameters were recorded, i.e., left ventricular end-diastolic diameter indexed to body surface area (BSA), interventricular septal thickness, posterior wall thickness, amplitude of interventricular septal motion, amplitude of posterior wall motion, EF, FS, the ratio of early to late diastolic mitral inflow velocities (E/A ratio), and the severity of mitral and tricuspid regurgitation. The wall thickness and ventricular dimensions derived from echocardiographic measurements were converted into z-scores using the Boston z-score calculator [12,13]. Treatment details for all children were recorded, including the use of digoxin, vasoactive drugs, diuretics, angiotensin-converting enzyme inhibitors (ACEIs)/angiotensin II receptor blockers (ARBs), β-blockers, intravenous immunoglobulin for intravenous injection (IVIG), glucocorticoids, antiplatelets or anticoagulants, and noninvasive or invasive respiratory support.

### 2.3. Follow-Up and Grouping

All patients were managed with standard heart failure pharmacotherapy and discharged with structured follow-up protocols, including inpatient review, outpatient clinic visits, or telephone consultations. The follow-up endpoints were defined as the occurrence of all-cause mortality (including documented cardiovascular death, non-cardiovascular death, and unexplained sudden death) or heart transplantation.

### 2.4. Statistical Methods

In the present study, all statistical analyses were conducted using SPSS version 26.0 (IBM, New York, NY, USA) and R software (version number: 4.2.0, Fig. http://www.R-project.org). For the limited instances of missing data, we employed mean/median/mode imputation based on the statistical characteristics of the available data. For continuous variables, group comparisons were conducted using either Student’s *t*-test or the Mann–Whitney U test. Categorical variables were compared using the χ^2^ test. To identify potential predictors of death or heart transplantation in children with DCM, we screened variables with a *p*-value < 0.10 between the D/HT group and the non-D/HT group. Variables exhibiting multicollinearity were excluded from further analysis. Binary logistic regression analysis was then performed to model the risk of death or heart transplantation. Nomogram models were developed based on the logistic regression results. Each variable was assigned a corresponding score according to its regression coefficient and odds ratio obtained from the logistic regression model. A predictive scoring system was developed to assign cumulative scores to each participant. The ROC curve evaluated the discriminatory power of both the nomogram and scoring system, while the Hosmer–Lemeshow (HL) test assessed the calibration by comparing the predicted and observed outcomes, yielding an adjusted C-index. Internal validation via bootstrap resampling minimized overfitting. Sensitivity and specificity were calculated based on the optimal cut-off from the maximum Youden index, ensuring a concise and robust model.

## 3. Results

### 3.1. General Information

A total of 233 patients, enrolled between January 2019 and June 2024, were included in this study. After a follow-up at 35.73 ± 43.71 months, 45 patients (19.31%) either died or underwent heart transplantation, including 36 patients (15.45%) who died of all causes and 9 patients (3.86%) who underwent heart transplantation.

### 3.2. Establishment and Test of Prediction Model

#### 3.2.1. Univariate Analysis

The D/HT group had a higher age of onset (*p* < 0.001), a higher proportion of cardiac functional classification III-IV (*p* < 0.001), a higher susceptibility to fatigue (*p* = 0.007), and a higher susceptibility to edema (*p* = 0.096) than the non-D/HT group. In the D/HT group, high-sensitivity troponin (*p* = 0.010), BNP or NTproBNP (as multiples of the upper limit of normal; *p* = 0.001), hemoglobin (*p* = 0.029), serum creatinine (*p* < 0.001), serum urea nitrogen (*p* = 0.001), serum uric acid (*p* < 0.001), ALT (*p* = 0.011), serum direct bilirubin (*p* < 0.001), and free T4 (*p* < 0.001) were higher than those in the non-D/HT group; however, AST (P = 0.084), serum calcium (*p* < 0.001), and free T3 (*p* = 0.091) were lower than those in the non-D/HT group. The D/HT group had a higher proportion of moderate or massive mitral regurgitation (*p* < 0.001) and a higher proportion of moderate or massive tricuspid regurgitation (*p* = 0.067) than the non-D/HT group, and the D/HT group had a smaller interventricular septal motion amplitude (*p* = 0.059), lower EF% (*p* < 0.001), and lower FS% (*p* < 0.001) than the non-D/HT group. The D/HT group was more likely to have atrioventricular block (*p* = 0.060), pathological Q waves (*p* = 0.025), ventricular extrasystoles (*p* = 0.008), ventricular tachycardia (*p* = 0.036), and low voltage in limb leads (*p* < 0.001) than the non-D/HT group, and the mean 24 h Holter heart rate was lower in the D/HT group than in the non-D/HT group (*p* = 0.056). The D/HT group had a higher proportion of patients treated with digoxin (*p* = 0.076) and vasoactive drugs (*p* = 0.001) and a lower proportion treated with β-blockers (*p* = 0.024) than the non-D/HT group; see Table 1 for specific results.

#### 3.2.2. Collinearity Diagnosis

Based on the results of the univariate analysis, variables with p-values less than 0.1 were selected for further collinearity diagnostics. The analysis revealed significant collinearity between EF (tolerance = 0.047, VIF = 21.074) and FS (tolerance = 0.056, VIF = 17.924). Considering its clinical significance, EF was retained as a predictor. After this adjustment, a re-evaluation of collinearity showed no significant multicollinearity among the remaining variables.

#### 3.2.3. Multivariate Analysis and Establishment of Nomogram and Scoring System Models

Twenty-nine variables that exhibited statistically significant differences and displayed no evidence of multicollinearity in the preceding univariate analysis were subsequently incorporated into the binary logistic regression model. The results showed the age at onset (OR = 0.993, 95% CI: 0.986–1.000), cardiac functional classification III-IV (OR = 16.796, 95% CI: 1.399–201.581), presence of moderate-to-massive mitral regurgitation (OR = 2.585, 95% CI: 1.078–6.201), presence of low voltage in limb leads on the electrocardiogram (OR = 4.252, 95% CI: 1.050–17.225), and need for vasoactive drugs (OR = 2.773, 95% CI: 1.037–7.417) were independent factors for the risk of death/heart transplantation in children with DCM; see Table 2. Meanwhile, we conducted the subgroup analysis of the aforementioned indicators between the death group and the heart transplantation group, and there were no statistically significant differences between the two groups (see Supplementary Table S1).

**Figure 1:**
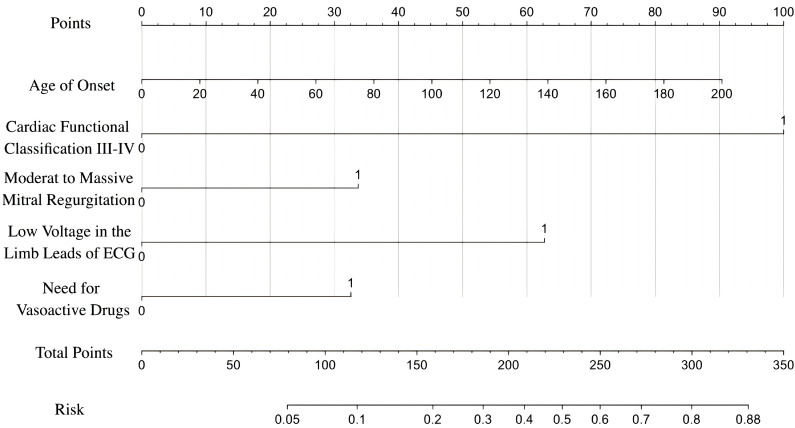
Nomogram for predicting the risk of death/heart transplantation in children with DCM. To predict the risk of death/heart transplantation, the patient score for each axis is marked, a line perpendicular to the point axis is drawn, and the points for all variables are summed. 0 represents no such manifestation, and 1 represents the presence of such manifestation. Next, the sum is marked on the total point axis and a line perpendicular to the probability axis is drawn. ECG, electrocardiogram; DCM, dilated cardiomyopathy.

A predictive nomogram model, designed to estimate the risk of mortality and the need for heart transplantation in pediatric patients with DCM, was meticulously developed using binary logistic regression analysis (Figure 1). For each patient, a perpendicular line was drawn from the fractional axis based on several prognostic factors: the age at onset, cardiac functional classification III-IV, presence of moderate-to-large mitral regurgitation, low voltage in limb leads on an ECG, and need for vasoactive drugs. The scores corresponding to each of these factors were then summed. This total score was plotted on the sum score axis, and a perpendicular line was extended to the probability axis to determine the predicted probability of mortality or heart transplantation. The predictive model demonstrated an older age at onset, cardiac functional class III-IV, moderate-to-severe mitral regurgitation, low voltage in limb leads on ECG, and need for vasoactive drugs prior to treatment were significant predictors of increased risk for mortality or heart transplantation in pediatric patients with DCM.

In the predictive model, the variable with the smallest absolute OR—age at onset—was designated as the reference. Scores were assigned to each variable based on their respective ORs and regression coefficients, as detailed in Table 2: (1) age of onset: 1 point if the age of onset was ≥ 58.5 months, and 0 points otherwise; (2) cardiac functional classification: if the cardiac functional classification was grade III-IV, 10 points were obtained; otherwise, 0 points were obtained; (3) mitral regurgitation: 2.5 points if the mitral valve demonstrated moderate-to-massive regurgitation, and 0 points otherwise; (4) low voltage in limb leads: 4 points if there was low voltage in limb leads on the electrocardiogram, and 0 points otherwise; and (5) vasoactive drugs: 3 points if vasoactive drugs were required, and 0 points otherwise. The total score for each patient, summing all five variables, ranged from 0 to 20.5 points.

#### 3.2.4. Test of Nomogram Model and Scoring System Model

**Figure 2:**
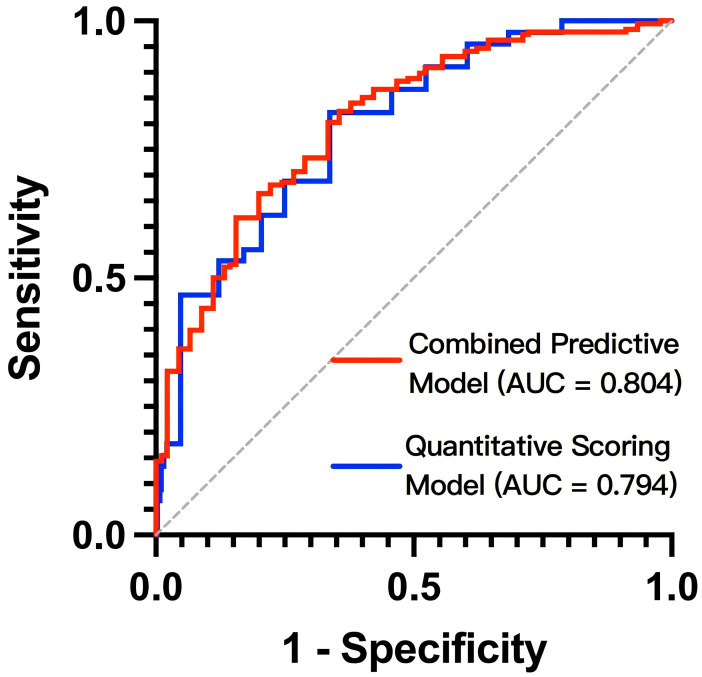
The ROC curves of models in predicting the risk of death/heart transplantation in children with DCM. The y-axis represents the sensitivity to predict the risk of death/heart transplantation; the x-axis represents the specificity to predict the risk of death/heart transplantation. The 45-degree reference line of the chart indicates that the sensitivity and the specificity are equal. ROC, receiver operating characteristic curve; AUC, area under the curve; DCM, dilated cardiomyopathy.

**Figure 3:**
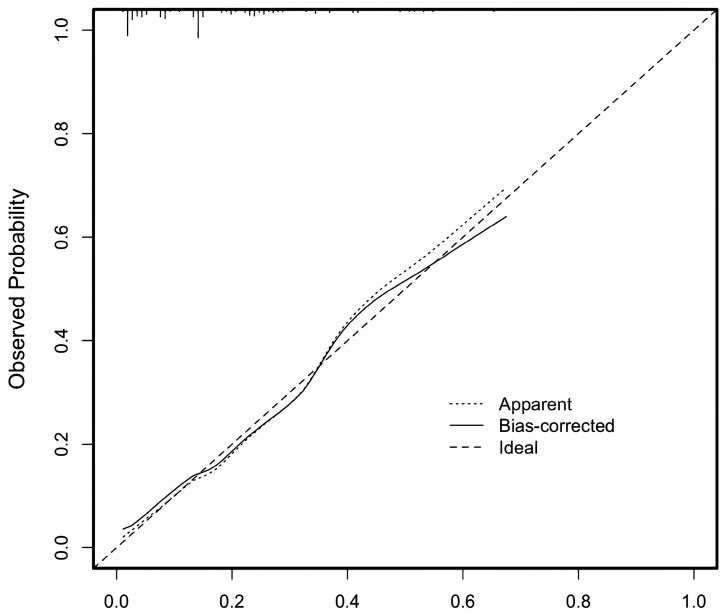
The calibration of the nomogram for predicting the risk of death/heart transplantation in children with dilated cardiomyopathy.

The ROC curve generated from the nomogram model was depicted in Figure 2, with an AUC of 0.804 (95% CI: 0.734–0.875), demonstrating a sensitivity of 80.3% and a specificity of 66.7% for predicting the risk of mortality or heart transplantation in pediatric patients with DCM. The corrected C-index of this prediction model was 0.79, and the H-L test yielded χ^2^ = 0.51238, *p* = 0.774 > 0.05. For internal validation, bootstrap resampling was employed, achieving an accuracy of 81.2%. The results indicate that the prediction model exhibits strong concordance with actual observations and does not exhibit overfitting (see Figure 3).

**Figure 4:**
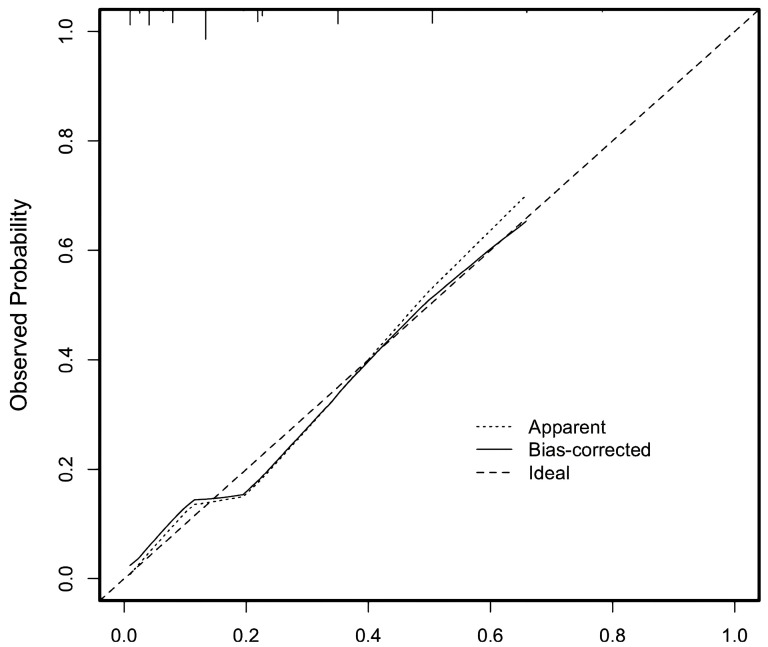
The calibration of the scoring system for predicting the risk of death/heart transplantation in children with dilated cardiomyopathy.

The total score for each child with DCM was calculated, and ROC curve analysis was performed to evaluate the predictive performance of the scoring system model. The results demonstrated an AUC of 0.794 (95% CI: 0.724–0.864; P < 0.001), as illustrated in Figure 2. The data did not show overfitting (see Figure 4). As shown in Table 3, the Youden index reached its maximum value at a total score threshold of 13.25, yielding a sensitivity of 68.9% and a specificity of 73.9% for predicting the risk of death or heart transplantation in pediatric patients with DCM.

## 4. Discussion

This study demonstrated that several factors can be utilized to predict the risk of mortality or heart transplantation in pediatric patients with DCM. These factors include the age of onset, cardiac functional classification III-IV, moderate-to-massive mitral regurgitation, low voltage in limb leads on ECG, and need for vasoactive drugs. The combined use of these predictors significantly enhances the accuracy of risk prediction. Consequently, a nomogram prediction model along with a scoring system was developed.

DCM represents a myocardial disorder of indeterminate etiology potentially associated with genetic predisposition, immune-mediated processes, and viral infections. It constitutes a prevalent cause of heart failure and sudden cardiac death, and is the leading indication for cardiac transplantation [4]. Recent studies have shown that the 5-year mortality rate of children with DCM is approximately 15–25% [7]. Our investigation revealed a 19.3% incidence of mortality or heart transplantation in pediatric patients with DCM over a three-year follow-up period, underscoring the persistently poor prognosis in this population despite conventional pharmacotherapy for heart failure. Numerous randomized, placebo-controlled clinical trials have demonstrated that specific medications can decrease hospitalization rates and mortality in adult heart failure cases [14]. These pharmacological agents include the angiotensin receptor/neprilysin inhibitor (ARNI) sacubitril/valsartan [15]; ivabradine [16]; the sodium–glucose cotransporter 2 (SGLT2) inhibitors empagliflozin [17] and dapagliflozin [18]; the soluble guanylate cyclase stimulator vilexiguat [19]; the specific cardiac myosin activator omecamtiv mecarbil [20], etc. Notably, analogous large-scale studies have not yet been reported in the pediatric population [9]. As novel therapeutic agents for heart failure, supported by robust evidence-based medicine, are increasingly utilized, the prognosis for children with DCM is anticipated to improve significantly.

In this study, the D/HT group exhibited a significantly higher mean age of onset and more severe cardiac dysfunction compared to the non-D/HT group. An age at onset exceeding 58.5 months and cardiac functional classification of III-IV were identified as predictors of mortality or heart transplantation in pediatric patients with DCM. Previous studies have demonstrated that the age of onset in children with DCM is closely associated with the prognosis, with a higher mortality rate and poorer outcomes observed in those diagnosed at an older age [21,22,23]. These findings are consistent with the present study. Furthermore, an analysis of 741 children under the age of 18 with DCM [24] revealed that younger survivors exhibited the highest incidence of normalized left ventricular size and function at initial diagnosis, as assessed by echocardiography. In contrast, only 10% of children aged over 10 years at presentation showed normalized left ventricular dimensions and function at the two-year follow-up period. Additionally, Lewis et al. found that children with DCM who achieved the normalization of cardiac function tended to be younger than those with persistent cardiac dysfunction [25], underscoring the critical role of age at onset in prognostic assessments for pediatric DCM. The NYHA and Ross functional classification systems are among the most widely utilized tools for assessing cardiac function in clinical practice, as their predictive value is well established. According to Ahmed A, heart failure patients classified as NYHA class III-IV exhibit significantly higher mortality and rehospitalization rates compared to those categorized as NYHA class I-II [26]. Muntwyler J et al. [27] followed 411 patients, and 68 patients died, with an overall mortality rate of 12.6%, including 7.1%, 15.0%, and 28.0% among NYHA class II, III, and IV patients, respectively. However, the subjective nature of physician judgment in assigning cardiac functional classification can be influenced by factors such as personal experience and clinical bias [28]. Therefore, in this study, cardiac functional classification was combined with other indicators to improve its predictive efficacy. We also observed that a positive family history was not identified as a risk factor for adverse cardiovascular events in children with DCM. This finding may be attributed to the inherent limitations of retrospective studies, or a de novo mutation in the children with DCM in our study cohort. Additionally, a positive family history might encourage family members to undergo cardiac screening, leading to earlier diagnosis and timely follow-up and intervention [29].

Children with DCM may develop mitral regurgitation secondary to diffuse myocardial injury. This condition leads to multiventricular chamber enlargement; structural alterations in the ventricular geometry; displacement of the papillary muscles, which in turn pull on the mitral leaflets; and dilation of the mitral annulus. In this investigation, we observed that moderate-to-severe mitral regurgitation significantly elevates the risk of adverse cardiovascular events. Consistent with previous studies, our findings suggest that mitral regurgitation is correlated with heightened symptom severity and an increased incidence of hospitalization in individuals with chronic heart failure [30,31,32]. The underlying mechanism may involve the chronic effects of mitral regurgitation, which exacerbate ventricular remodeling through the activation of matrix metalloproteinases, leading to the degradation of the extracellular matrix and the release of numerous neurohormones and cytokines [33]. Our study revealed that a reduced QRS voltage in limb leads on an ECG can serve as a potential predictor for adverse cardiovascular events in pediatric patients with DCM. Patchy myocardial fibrosis is a hallmark of DCM and has been implicated by several scholars as the primary factor contributing to a reduced QRS voltage on an ECG [34]. Recent evidence demonstrates that the amplitude of the QRS complex on a surface ECG diminishes in patients with chronic heart failure and increases with clinical improvement, suggesting that QRS amplitude may serve as a valuable indicator of therapeutic response in heart failure management [35,36]. The findings of this study underscore the significance of routine monitoring through echocardiography and an ECG in children with DCM. Clinicians should closely evaluate the severity of mitral regurgitation and changes in limb lead voltage in these patients. Such assessments enable more precise individualization of treatment strategies and enhance the ability to predict patient outcomes accurately. To evaluate myocardial fibrosis, we attempted to collect patients’ cardiac MRI data. Regrettably, only 1/5 of the patients completed cardiac MRI, which was constrained by multiple factors, including young age (making it difficult to tolerate prolonged scans), disease severity, parental cooperation, and financial limitations. Among the patients who underwent cardiac MRI, the majority exhibited findings such as left ventricular enlargement and reduced EF, and approximately half of them showed evidence of myocardial late gadolinium enhancement, consistent with the presence of patchy myocardial fibrosis.

The American Heart Association (AHA) classification of Stage D heart failure refers to refractory heart failure that remains symptomatic despite optimal medical therapy. Patients in this stage may benefit from advanced specialized interventions, such as mechanical circulatory support or continuous intravenous inotropic therapy [9]. According to the guidelines, early pharmacological intervention is recommended to target ventricular remodeling in patients with DCM. Medications such as β-blockers, ACEIs/ARBs, and MRAs can mitigate myocardial injury. In some cases, treatment may lead to a reversal of cardiac dysfunction and structural abnormalities, significantly improving the prognosis for patients with DCM [11]. In this study, we observed a higher prevalence of vasoactive drug utilization in the D/HT group compared to the non-D/HT group. This finding may be attributed to a greater proportion of patients with refractory heart failure requiring continuous intravenous inotropic support, which is associated with a higher mortality rate. Additionally, while multiple large-scale trials have demonstrated the survival benefits of β-blockers extending from mild heart failure (NYHA class II) to severe heart failure (NYHA class IV), the clinical application of β-blockers in pediatric patients with advanced chronic heart failure remains relatively uncommon [37,38,39,40]. This discrepancy likely explains the lower frequency of β-blocker use in the D/HT group relative to the non-D/HT group. Further investigation and more robust data are necessary to elucidate the potential benefits of β-blockers in this specific patient population.

In this study, we developed both a nomogram model and a scoring system model with high predictive accuracy to address the limitations of relying on single predictive factors, which inherently possess limited prognostic value. The nomogram model transforms complex regression equations into an intuitive graphical format, while the scoring system simplifies variable settings, making it more accessible and easier to implement in clinical practice. These tools can assist healthcare providers in assessing and selecting appropriate treatment strategies for children with DCM, ultimately facilitating personalized medicine approaches. However, this study has certain limitations. As a single-center retrospective analysis, it is subject to inherent design constraints. The evaluated performance of the scoring system model is relatively low. One potential explanation is that the limited sample size of the current study may have constrained the model’s capacity to capture the full spectrum of disease heterogeneity, thereby potentially reducing its discriminative power. This study did not include genetic testing and cardiac MRI data as primary predictors due to incomplete genetic testing (constrained by patient preferences) and low MRI completion rates (attributed to young age, disease severity, parental cooperation, and financial limitations). Despite these limitations, future research should include multicenter studies with larger sample sizes to further validate these findings and enhance their generalizability.

In summary, the risk of mortality and the necessity for heart transplantation in pediatric patients with DCM are independently associated with multiple factors. Strengthened treatment and follow-up for patients exhibiting these risk factors, guided by an individualized therapeutic approach, can help reduce mortality and improve prognosis in children with DCM.

## Figures and Tables

**Table 1:** Comparison of characteristics between D/HT and non-D/HT groups.

Factors Groups			D/HT	Non-D/HT	t/Z/χ^2^ Value	*p*-Value
General Data and Clinical Manifestations	Number [n (%)]		45 (19.3%)	188 (80.7%)	-	-
	Sex (M/F)		24/21	95/93	0.114	0.736
	Age (months)		90.5 (38.0, 141.0)	44.5 (8.0, 101.5)	−3.632	<0.001
	BMI (kg/m^2^)		17.18 (14.60, 22.13)	16.06 (14.15, 19.21)	−1.363	0.173
	Diagnosis Time (months)		1.50 (0.38, 12.0)	1.00 (0.50, 2.25)	−0.108	0.914
	Dyspnea [n (%)]		16 (35.6%)	74 (39.4%)	0.222	0.638
	Fatigue [n (%)]		43 (95.6%)	147 (78.2%)	7.275	0.007
	Edema [n (%)]		14 (31.1%)	37 (19.7%)	2.775	0.096
	Syncope or Presyncope [n (%)]		2 (4.4%)	10 (5.3%)	0.057	0.812
	Cardiac Arrest [n (%)]		0 (0%)	4 (2.1%)	0.974	0.324
	Growth Retardation [n (%)]		4 (8.9%)	14 (7.4%)	0.106	0.745
	NYHA/Ross Class [n (%)]	I-II	2 (4.4%)	64 (34.0%)	15.667	<0.001
		III-IV	43 (95.6%)	124 (66.0%)		
	Family History of DCM [n (%)]		9 (20%)	22 (11.7%)	2.167	0.141
	Follow-up Time (months)		43.33 ± 19.21	33.90 ± 47.60	1.302	0.194
Blood Analysis Tests	CK (U/L)		81.50 (50.50, 112.50)	79.50 (55.75, 143.25)	−0.887	0.375
	CK-MB Mass Assay (ng/mL)		2.41 (1.70, 3.18)	2.02 (1.08, 3.67)	−1.126	0.260
	hs-cTnI (ng/mL)		0.029 (0.005, 0.279)	0.004 (0.001, 0.016)	−2.586	0.010
	Multiple of BNP or NTproBNP Above Upper Limit of Normal		15.02 (4.07, 21.85)	2.15 (0.22, 15.72)	−3.376	0.001
	HGB (g/L)		126.93 ± 17.22	120.44 ± 17.97	2.194	0.029
	SCR (umol/L)		51.20 (39.30, 64.75)	32.00 (26.15, 47.68)	−5.007	<0.001
	BUN (mmol/L)		6.76 ± 4.00	5.04 ± 2.78	3.407	0.001
	UA (umol/L)		426.40 (285.63, 550.35)	304.05 (249.50, 412.43)	−3.795	<0.001
	ALT (U/L)		19.00 (13.30, 48.10)	18.80 (13.25, 31.15)	−2.535	0.011
	AST (U/L)		33.45 (25.55, 68.90)	33.85 (23.70, 46.05)	−1.727	0.084
	TBIL (umol/L)		17.47 ± 8.31	17.48 ± 27.71	−0.003	0.998
	DBIL (umol/L)		2.21 (1.46, 3.47)	1.50 (0.96, 2.39)	−3.904	<0.001
	IBIL (umol/L)		13.76 ± 6.63	13.55 ± 23.41	0.057	0.954
	Serum Potassium (mmol/L)		4.27 ± 0.50	5.11 ± 9.66	−0.580	0.563
	Serum Sodium (mmol/L)		136.90 (135.85, 138.00)	136.35 (134.40, 137.90)	−1.139	0.255
	Serum Calcium (mmol/L)		2.29 (2.20, 2.42)	2.39 (2.31, 2.48)	−3.680	<0.001
	TSH (mIU/L)		2.21 (0.90, 3.57)	2.63 (1.35, 3.54)	−0.118	0.906
	T3 (nmol/L)		1.93 (0.88, 89.92)	1.77 (1.37, 65.33)	−0.267	0.790
	T4 (nmol/L)		97.30 (8.90, 118.28)	105.70 (10.16, 132.48)	−0.883	0.377
	FT3 (pmol/L)		4.97 ± 1.03	5.30 ± 1.16	−1.697	0.091
	FT4 (pmol/L)		15.55 ± 3.75	13.26 ± 3.64	3.636	<0.001
	BG (mmol/L)		5.71 ± 0.92	5.55 ± 1.28	0.831	0.407
	Serum Vitamin D (nmol/L)		51.29 (38.30, 72.66)	48.51 (37.00, 64.60)	−0.559	0.576
	Hcy (umol/L)		9.11 ± 3.46	9.06 ± 4.41	0.052	0.958
Echocardiography	Z-score Value of IVSd		−2.11 (−2.63, −1.99)	−2.31 (−2.86, −1.96)	−1.412	0.158
	IVSE (mm)		4.55 (3.93, 5.15)	4.60 (3.48, 5.50)	−1.886	0.059
	Z-score Value of LVEDD		6.20 ± 2.66	5.58 ± 3.47	1.323	0.190
	Z-score Value of LVPWd		−2.04 ± 1.54	−2.10 ± 0.79	0.404	0.687
	PWE (mm)		5.40 ± 2.67	5.79 ± 3.03	−0.789	0.431
	EF (%)		30.5 ± 14.0	39.2 ± 14.8	−3.560	<0.001
	FS (%)		13.0 (10.0, 21.5)	19.5 (14.8, 31.0)	−4.023	<0.001
	E/A		1.60 (1.33, 1.98)	1.50 (1.30, 1.70)	−1.091	0.275
	MR [n (%)]	Mild	18 (40.0%)	133 (70.7%)	15.048	<0.001
		Moderate or Severe	27 (60.0%)	55 (29.3%)		
	TR [n (%)]	Mild	36 (80.0%)	169 (89.9%)	3.361	0.067
		Moderate or Severe	9 (20%)	19 (10.1%)		
Electrocardiogram	Conduction Block [n (%)]		20 (44.4%)	56 (29.8%)	3.549	0.060
	Pathological Q Waves [n (%)]		4 (8.9%)	4 (2.1%)	5.006	0.025
	Prolonged QT Interval [n (%)]		3 (6.7%)	16 (8.5%)	0.165	0.685
	ST/T Abnormalities [n (%)]		41 (91.1%)	152 (80.9%)	2.688	0.101
	Atrial Premature Contraction [n (%)]		22 (48.9%)	67 (35.6%)	2.701	0.100
	Ventricular Premature Contraction [n (%)]		31 (68.9%)	88 (46.8%)	7.084	0.008
	Atrial Tachycardia [n (%)]		1 (2.2%)	11 (5.9%)	0.979	0.322
	Ventricular Tachycardia [n (%)]		9 (20%)	17 (9.0%)	4.397	0.036
	High QRS Voltage [n (%)]		13 (28.9%)	61 (32.4%)	0.212	0.645
	Low QRS Voltage [n (%)]		9 (20%)	7 (3.7%)	15.041	<0.001
	Average Heart Rate (bpm)		98.6 ± 21.0	106.3 ± 22.5	−1.920	0.056
	SDNN (ms)		98.90 (58.00, 136.25)	98.50 (74.75, 126.00)	−0.685	0.493
	SDANN (ms)		84.88 ± 43.47	84.02 ± 38.18	0.123	0.903
	RMSSD (ms)		26.50 (13.25, 36.75)	30.00 (18.00, 45.50)	−1.082	0.279
	pNN50 (%)		4.77 (0.70, 16.02)	7.08 (1.62, 20.06)	−0.776	0.438
Treatment	Digoxin [n (%)]		41 (91.1%)	150 (79.8%)	3.151	0.076
	Vasoactive Agents [n (%)]		32 (71.1%)	80 (42.6%)	11.862	0.001
	Diuretics [n (%)]		42 (93.3%)	158 (84.0%)	2.578	0.108
	ACEI/ARB [n (%)]		44 (97.8%)	180 (95.7%)	0.404	0.525
	β-blockers [n (%)]		5 (11.1%)	51 (27.1%)	5.102	0.024
	IVIG [n (%)]		9 (20.0%)	49 (26.1%)	0.714	0.398
	Glucocorticoids [n (%)]		25 (55.6%)	97 (51.6%)	0.228	0.633
	Aspirin [n (%)]		11 (24.4%)	41 (21.8%)	0.146	0.703
	Anticoagulants [n (%)]		2 (4.4%)	13 (6.9%)	0.368	0.544
	Noninvasive or Invasive Respiratory Support [n (%)]		3 (6.7%)	24 (12.8%)	1.318	0.251

DCM, dilated cardiomyopathy; D/HT, death/heart transplantation; M/F, male/female; BMI, body mass index; kg/m^2^, kilogram per square meter; NYHA, New York Heart Association; SCD, sudden cardiac death; CK, creatine kinase; BNP, B-type natriuretic peptide; NTproBNP, N-terminal pro brain natriuretic peptide; HGB, hemoglobin; SCR, serum creatinine; BUN, blood urea nitrogen; UA, uric acid; ALT, alanine aminotransferase; AST, aspartate aminotransferase; TBIL, total bilirubin; DBIL, direct bilirubin; IBIL, indirect bilirubin; TSH, FT3, thyrotropin; T3, triiodothyronine; T4, thyroxine; FT3, free triiodothyronine; FT4, free thyroxine; BG, blood glucose; Hcy, homocysteine; IVSd, interventricular septal thickness at diastole; IVSE, interventricular septum excursion; LVEDD, left ventricular end-diastolic diameter; LVPWd, left ventricular posterior wall thickness at diastole; PWE, posterior wall excursion; EF, ejection fraction; FS, fractional shortening; MR, mitral regurgitation; TR, tricuspid regurgitation; bpm, beats per minutes; SDNN, standard deviation of normal to normal intervals; SDANN, standard deviation of the average normal to normal intervals; RMSSD, root mean square of successive differences; pNN50, percentage of adjacent normal to normal intervals differing by more than 50 ms; ACEI/ARB, angiotensin-converting enzyme inhibitors/angiotensin II receptor blockers; IVIG, intravenous immunoglobulin.

**Table 2:** Coefficients of binary logistic regression for predicting risk of death/heart transplantation in children with DCM.

Variable(s)	B	S.E.	Wald	*p*-Value	OR	95% CI for OR	Points
Age	−0.008	0.004	4.362	0.037	0.993	0.986–1.000	1
NYHA/ROSS Class	2.821	1.268	4.951	0.026	16.796	1.399–201.581	10
MR	0.950	0.446	4.526	0.033	2.585	1.078–6.201	2.5
Low QRS Voltage	1.447	0.714	4.113	0.043	4.252	1.050–17.225	4
Digoxin	−2.380	1.254	3.604	0.058	0.093	0.008–1.080	-
Vasoactive Agents	1.020	0.502	4.130	0.042	2.773	1.037–7.417	3
Constant	−0.502	0.797	0.398	0.528	0.605	-	-

DCM, dilated cardiomyopathy; NYHA, New York Heart Association; MR, mitral regurgitation; S.E., standard error; OR, odds ratio; CI, Confidence Interval.

**Table 3:** The cut-off value of variables predicting the risk of death/heart transplantation in children with DCM.

Variable(s)	AUC	95% CI	*p*-Value	Cut-off Value	Sensitivity	Specificity
Age	0.674	0.594–0.755	<0.001	≥58.5 months	66.7%	63.3%
NYHA/ROSS Class	0.648	0.570–0.726	0.002	≥0.5	95.6%	34.0%
MR	0.654	0.562–0.745	0.001	≥0.5	60.0%	70.7%
Low QRS Voltage	0.581	0.482–0.681	0.090	≥0.5	20.0%	96.3%
Vasoactive Agents	0.643	0.555–0.731	0.003	≥0.5	71.1%	57.4%
Combined Predictive Model	0.804	0.734–0.874	-	-	80.3%	66.7%
Quantitative Scoring Model	0.794	0.724–0.864	<0.001	≥13.25	68.9%	73.9%

DCM, dilated cardiomyopathy; AUC, area under receiver operating characteristic curve; CI, Confidence Interval; NYHA, New York Heart Association; MR, mitral regurgitation.

## Data Availability

Data from this study are available upon approval by emailing the corresponding author.

## Supplementary Materials

The following supporting information can be downloaded at: https://www.mdpi.com/article/10.3390/children12070880/s1, Table S1: Differences in predictors between death group and heart transplantiation group in children with DCM.
