# Supplementary material for: Prognosis of Pediatric Dilated Cardiomyopathy: Nomogram and Risk Score Models for Predicting Death/Heart Transplantation
Source: Children (Basel). 2025 Jul 3;12(7):880. doi: 10.3390/children12070880 (PMC12293697; doi:10.3390/children12070880)
Supplement: Supplementary file 1 [file children-12-00880-s001.zip › children-3649557-supplementary.pdf]

Supplementary Table S1. Differences in predictors between death group and heart transplantiation group in children with DCM.

Factors<br>Groups	Age<br>(months)	NYHA/ROSS class	MR	Low QRS Voltage	Vasoactive agents
Death	94.5<br>(37.8,138.8)	9 (100%)	5<br>(55.6%)	2 (22.2%)	6 (66.7%)
Heart Transplantation	72.0<br>(23.0,144.0)	34 (94.4%)	22<br>(61.1%)	7 (19.4%)	26 (72.2%)
Z/χ2 value	-0.014	0.523	0.093	0.035	0.108
P value	0.989	0.469	0.761	0.852	0.742

DCM, dilated cardiomyopathy; MR, Mitral regurgitation; NYHA, New York Heart Association
